# Differences in empathy toward patients between medical and nonmedical students: an fMRI study

**DOI:** 10.1007/s10459-021-10045-y

**Published:** 2021-04-20

**Authors:** Shin Ah Kim, Young-Mee Lee, Stephan Hamann, Sang Hee Kim

**Affiliations:** 1 Department of Brain and Cognitive Engineering, Korea University, 145 Anam-ro, Seongbuk-gu, Seoul, 02841 South Korea; 2 Department of Medical Education, Korea University College of Medicine, Seoul, South Korea; 3 Department of Psychology, Emory University, Atlanta, GA USA

**Keywords:** Cognitive empathy, Affective empathy, Patient, Medical students, fMRI

## Abstract

There is growing concern about a potential decline in empathy among medical students over time. Despite the importance of empathy toward patients in medicine, it remains unclear the nature of the changes in empathy among medical students. Thus, we systematically investigated affective and cognitive empathy for patients among medical students using neuroscientific approach. Nineteen medical students who completed their fifth-year medical curriculum and 23 age- and sex-matched nonmedical students participated in a functional magnetic resonance imaging study. Inside a brain scanner, all participants read empathy-eliciting scenarios while adopting either the patient or doctor perspective. Brain activation and self-reported ratings during the experience of empathy were obtained. Behavioral results indicated that all participants reported greater emotional negativity and empathic concern in association with the patient perspective condition than with the doctor perspective condition. Functional brain imaging results indicated that neural activity in the posterior superior temporal region implicated in goal-relevant attention reorienting was overall increased under the patient perspective than the doctor perspective condition. Relative to nonmedical students, medical students showed decreased activity in the temporoparietal region implicated in mentalizing under the patient perspective versus doctor perspective condition. Notably, this same region showed increased activity under the doctor versus patient condition in medical students relative to nonmedical students. This study is among the first to investigate the neural mechanisms of empathy among medical students and the current findings point to the cognitive empathy system as the locus of the primary brain differences associated with empathy toward patients.

## Introduction

Empathy of physicians for their patients is associated with improved healthcare quality and patient outcomes as well as the well-being of physicians themselves (Decety & Fotopoulou, 2015; Underman & Hirshfield, 2016). On the other hand, empathizing with other’s pain and suffering can increase one’s own distress and, therefore, reduce the ability for empathy (Eisenberg & Eggum, 2009). For example, medical professionals, who are repeatedly exposed to distress and pain among patients in their everyday routine, have a greater risk of burnout and emotional disturbances (Adams et al., 2006; Krasner et al., 2009), and eventually experience reduced empathy as they gain professional experience in the field (Hojat et al., 2009; Neumann et al., 2011; Newton et al., 2008).

Evidence is mixed regarding whether empathy levels decrease among medical trainees over time with increased professional experience (Colliver et al., 2010; Costa et al., 2013; Hegazi et al., 2017; Kataoka et al., 2012; Smith et al., 2017). Some studies found no change in empathy (Costa et al., 2013; Hegazi et al., 2017), whereas others found an increase in empathy over the course of medical training (Kataoka et al., 2012; Smith et al., 2017). These mixed findings may be attributable to various factors, including the overreliance on self-reported assessments of empathy and inconsistent operational definitions of empathy across studies (Colliver et al., 2010; Pedersen, 2009; Sulzer et al., 2016). Empathy is a multifaceted psychological construct, and empathy tasks used in prior studies differ with regard to the extent to which they elicit different components of empathic response. For example, such tasks may elicit cognitive vs. affective aspects of empathy to varying degrees, and also in the extent to which emotional responses reflect empathic concern (i.e., emotions felt for the others) vs. self-oriented responses (e.g., focusing on one’s own feelings of distress; Batson, 2009). A social neuroscientific approach to empathy enables systematic investigation of the brain mechanisms that underlie these various aspects of empathy (Decety & Jackson, 2006; Shamay-Tsoory et al., 2009; Singer & Lamm, 2009).

A broadly accepted model of empathy in social neuroscience distinguishes between affective and cognitive components of empathy (Decety & Jackson, 2006; Shamay-Tsoory, 2011; Shamay-Tsoory et al., 2009; Zaki, 2014; Zaki & Ochsner, 2012). Affective empathy depends on perceptual simulation and emotional contagion processes which recruit brain regions involved in emotion perception and mirroring, such as the amygdala, anterior insula, anterior cingulate cortex, premotor cortex, pre- and post-central gyri, inferior frontal gyrus, and inferior parietal regions (Carr et al., 2003; Lindquist et al., 2012; Singer & Lamm, 2009). In contrast, cognitive empathy depends on the ability to adopt other people’s perspectives and make inferences about the mental states of others. Accordingly, this form of empathy recruits regions such as the temporoparietal junction (TPJ), superior temporal sulcus (especially its posterior part), temporal pole, precuneus/posterior cingulate gyrus, medial prefrontal cortex, and hippocampus (Frith & Frith, 2003; Mitchell, 2009; Shamay-Tsoory, 2011). In a clinical context, a physician’s affective empathy promotes feelings about the degree of pain and suffering a patient experiences, whereas a physician’s cognitive empathy promotes an intellectual understanding of the kind and quality of pain and suffering a patient experiences (Halpern, 2003; Hojat et al., 2009; Stepien & Baernstein, 2006).

Previous neuroimaging studies of empathy among medical professionals have reported reduced empathic neural responses to others’ pain associated with professional medical experience (Cheng et al., 2007; Decety et al., 2010). In an fMRI study, physicians with professional experience in acupuncture, relative to age-matched non-physician controls, showed reduced activity in brain regions specifically implicated in pain perception such as the anterior insula and anterior cingulate when observing photographs depicting needles being inserted into various body parts (Cheng et al., 2007). Physicians also reported feeling less pain and unpleasantness than did non-physician controls. Interestingly, activity in prefrontal regions implicated in executive control was also increased in physicians relative to controls, suggesting that physicians may engage explicit control or regulation to decrease their empathic responses toward others’ pain (Cheng et al., 2007). These neuroimaging findings are consistent with the view that empathic responses are flexible and can be modulated by cognitive and contextual factors, such as attention, situational appraisal, and adopted perspective (Singer & Lamm, 2009). These considerations suggest that investigating how behavioral and neural empathic responses are modified by such contextual factors will be necessary to better understand the nature of changes in empathy in medical professionals (Pedersen, 2009).

A recent neuroimaging study suggested that decreases in empathy among medical practitioners depend on the situational context (Cheng et al., 2017). In an fMRI study, professional nurses viewed photographs depicting injured body parts while imagining that it occurred to a friend or family member either in a hospital context or to the same person in a home context. Subjective ratings of valence and arousal to photographs depicting injury were obtained outside the scanner. Nurses exhibited reduced levels of activation in brain regions implicated in the affective sharing of pain, such as the anterior insula and anterior midcingulate cortex, when observing pain in the hospital context relative to the home context. In contrast, observing pain in the hospital context (as opposed to the home context) activated the right TPJ, a region known to be involved in the self-other distinction and mentalizing (Cheng et al., 2017). Nurses with longer work experience reported reduced emotional negativity and arousal when observing pain in the hospital context; but this relationship was not found in the home context. These findings suggest that experience-dependent desensitization of empathic responses to the pain of others is greater in the context matching that experience (i.e., the hospital context), and that the neural substrates of affective and cognitive empathy are differentially influenced by the situational context.

Although prior neuroimaging studies of empathy in medicine have contributed to our understanding of empathy among medical professionals, an important limitation of these studies is that these studies have typically examined empathic responses to individuals who are familiar to the subjects (Cheng et al., 2007, 2017; Decety et al., 2010). That is, the prior studies instructed participants (i.e., medical professionals) to imagine that pain was inflicted to close others such as friends or family members (Cheng et al., 2007, 2017; Decety et al., 2010). Thus, there is currently relatively little information about the neural mechanisms of empathic responses of medical professionals specifically toward patients. Professional medical organizations recognize empathy for patients as an essential aspect of patient care (Anderson et al., 1999; Krevans & Benson, 1983). Many medical schools include educational curriculum to promote empathy for patients (Patel, Pelletier-Bui, et al., 2019a). However, physician empathy is often understood as an intellectual understanding rather than an emotional sharing of the patient’s suffering (Hojat et al., 2009). It is valued as a means of ensuring reliable and efficient patient care and avoiding emotional burnout and compassion fatigue (Blumgart, 1964; Halpern, 2003, 2014; Hojat et al., 2009). This perspective may promote an ideal of the highly skilled, rational medical professional detached from their own emotions and those of their patients. Therefore, medical students, who adapt to a professional role while interacting with patients during clinical training may show qualitatively different empathic responses to patients as compared to non-patients. The brain correlates of these potential differences in empathy for patients in medical students can be systematically investigated using functional neuroimaging.

In the current study, we investigated neural mechanisms of empathy toward patients among medical students who had completed 1 year of their core clinical clerkship rotations in a hospital during the previous year. Nonmedical college students matched on key demographic characteristics (i.e., age, sex, years of education) participated as controls. To elicit empathic responses toward patient inside the brain scanner, we presented participants with short written anecdotal scenarios describing patient-doctor interactions in the clinic. The empathy version depicted a patient suffering from an illness and a doctor who was unconcerned about the patient. The neutral version depicted non-emotional routine interactions between a patient and a doctor. While reading each scenario, participants were specifically asked to adopt either the patient or doctor perspective, equally often across trials. Participants reported the emotional valence elicited by the scenario at the end of each trial. We expected that both medical and nonmedical students would show increased neural and behavioral measures of empathy while adopting the patient perspective relative to adopting the doctor perspective, as our scenarios detailed the illness and pain of the patients rather than that of the doctor. The key question of interest was whether the neural and behavioral measures of affective and cognitive empathy for the patient would be different between medical and nonmedical students. Given that affective empathy relies on emotion simulation that typically occurs automatically (Shamay-Tsoory, 2011), and cognitive empathy is sensitive to high-level cognitive and motivational factors (Shamay-Tsoory, 2011; Zaki, 2014), we expected that the main differences between groups would be manifested in cognitive empathy.

## Methods

### Participants

We recruited 19 medical students (six women, mean age = 24.53, standard deviation [SD] = 1.71) and 23 nonmedical students of matching age groups (seven women, mean age = 24.04, SD = 2.03) for the current fMRI study. Four participants from the nonmedical student group were eliminated from all data analyses due to excessive head movement (translation > 3.0 mm) during brain image acquisition. Therefore, 19 participants from each group were included in the data analysis. All participating medical students had completed their fifth-year medical curriculum just before their participation in this study. They had finished the core clinical clerkships of internal medicine, family medicine, surgery, pediatrics, obstetrics and gynecology, and radiology during the previous year. In contrast, all participating nonmedical students majored in engineering, business, language, or literature, and reported having no regular interaction with patients.

All participants were right-handed and without past or current neurological or psychiatric illnesses. This study was approved by the ethics committee of the institution and performed in accordance with the Declaration of Helsinki. For screening purposes, all participants completed the Spielberger State-Trait Anxiety Inventory (Spielberger, 1983) and the Beck Depression Inventory II (Beck et al., 1996) at the time of providing consent to participate in the study. Participants’ current moods were assessed using the Positive Affect and Negative Affect Scale (PANAS; Watson et al., 1988) right before the brain scan. Individual differences in trait empathy was assessed using the Interpersonal Reactivity Index (IRI), which consists of four subscales: personal distress, empathic concern, perspective taking and fantasy (Davis, 1980; Kang et al., 2009). The personal distress subscale and empathic concern subscale are considered to tap the emotional aspect of empathy and the perspective taking subscale and fantasy subscale are considered to tap the cognitive aspect of empathy (Harari et al., 2010).

### Development of empathy scenarios

Empathy scenarios were developed based on experience sampling with a separate group of medical students (n = 20) who had completed the final year of the medical curriculum. Specifically, sixth-year medical students volunteered to participate in individual interview sessions, wherein they were asked to freely recall clinical anecdotes during their clerkships when they felt empathy for patients and thought that it might have been helpful for a physician to show empathy. A total of 55 episodes were collected and sorted to produce 12 unique anecdotes. These anecdotes were dramatized and edited to be used as empathy-eliciting scenarios. Twelve neutral scenarios were also created describing routine patient-doctor interactions in the clinic without prominent emotional components. Each scenario was composed of three sentences. The first sentence introduced a patient’s visit to the hospital (e.g., “An elderly patient with Parkinson's disease was escorted by his wife to a neurologist”). The second sentence described the suffering of the patient (e.g., “He and his wife looked very nervous and puzzled with the doctor’s instructions”). Lastly, the third sentence described the responses of the doctor (e.g., “The doctor bluntly told them to wait outside for further instructions"). Neutral scenarios also included three sentences. Care was taken to best match the structure of the scenarios across the conditions (e.g., “An elderly person visited a hospital for a regular medical checkup. He said that he wanted an additional exam because of his smoking habit. The doctor asked if he wanted a CT scan on the chest”). The key differences between the empathy and neutral scenarios were best expressed in the second and third sentences. The length of each sentence was determined taking into account the typical reading speed and the time course (roughly, 14–16 s) of the fMRI hemodynamic response function (Boynton et al., 1996; Dale & Buckner, 1998).

### In-scanner empathy task

**Fig. 1:**
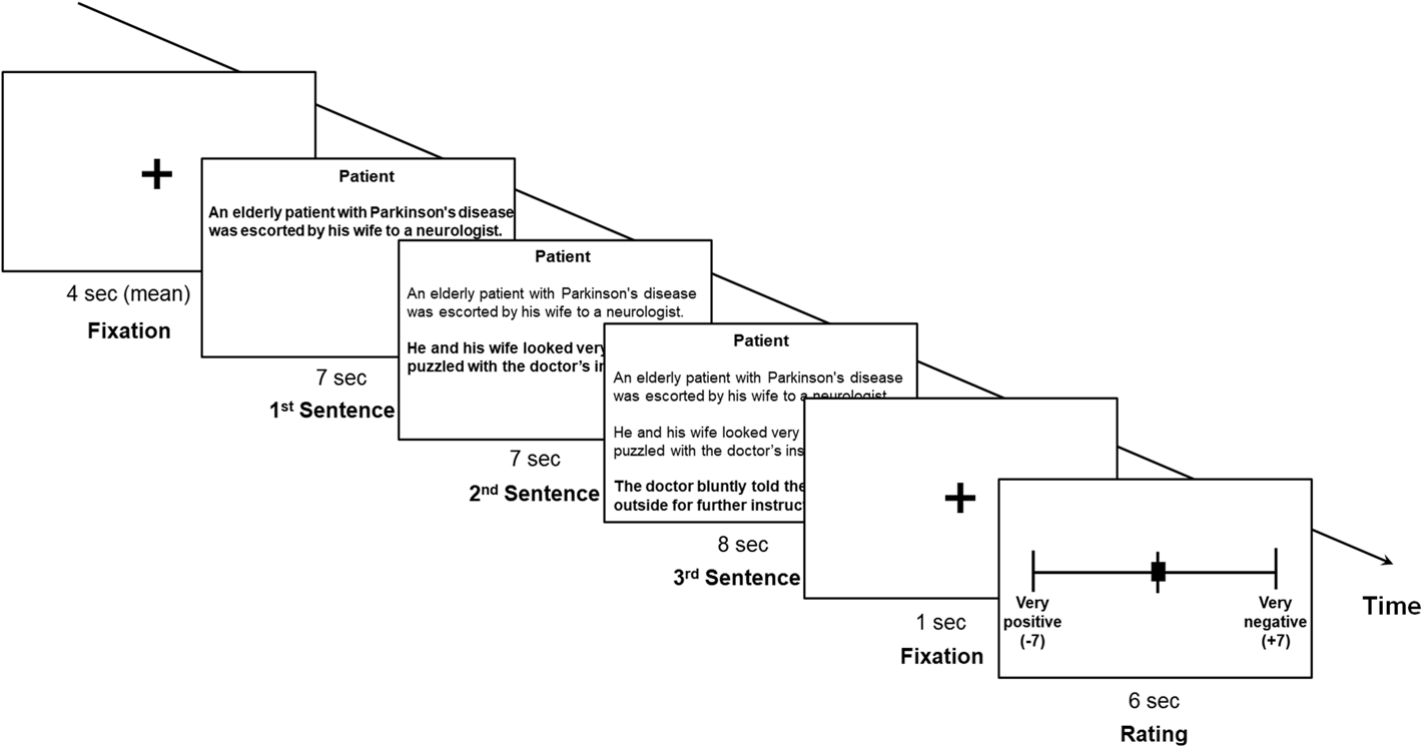
An illustration of the in-scanner empathy task showing a representative empathy-eliciting scenario trial. Each task trial started with a fixation cross, followed by each of the three sentences of the scenario, added sequentially. The first sentence introduced a patient’s visit to the hospital. The second sentence described the need or emotional state of the patient. The third sentence described the responses of the doctor. Next, following the brief presentation of a fixation cross, participants rated the emotional valence elicited by each scenario on a Likert scale ranging from − 7 (very positive) to + 7 (very negative)

Inside an fMRI brain scanner, participants were presented with a total of 24 scenarios and were asked to adopt either the patient or doctor perspective while reading each scenario. The participants performed a rating task consisting of two runs of four blocks each, with a 1-min break between the runs. Scenarios were presented in a blocked fashion so that six scenarios were presented consecutively with the same type of perspective condition. The presenting order of two perspective conditions was alternated between blocks. Each block consisted of three empathy scenarios and three neutral scenarios, presented in a pseudorandom order within the block. Each of the 24 scenarios was shown twice: once during the first run and again during the second run. A block started with an instructional cue of “doctor” or “patient” to indicate a perspective condition for the entire block. At the beginning of each task trial, a fixation cross appeared for either 3, 4, or 5 s (see Fig. 1). The first, second, and third sentences of the scenario were then presented sequentially, one at a time, each lasting 7, 7, and 8 s, respectively. After viewing the last sentence, participants rated the emotional valence elicited by each scenario on a Likert scale ranging from − 7 (very positive) to + 7 (very negative). Participants were instructed to complete the ratings within 6 s, and the next trial automatically followed.

Outside the scanner, participants were instructed to read each scenario one more time from the perspective of the patient and the doctor, respectively. Participants rated on a Likert scale of 1 to 7 (1, not at all; 7, very well) their success at adopting the perspectives of the target (patient or doctor) and empathic concerns (1, not at all; 7, very much) experienced for the target in accordance with their feelings while inside the scanner. Before the in-scanner task, a practice task of six trials with scenarios that were not presented during the in-scanner task was completed. One nonmedical student’s ratings of perspective-taking success and empathic concern were not recorded due to technical failures.

### Imaging data acquisition

Brain images were obtained at the Korea University Brain Imaging Center using a 3 T Siemens Trio scanner (Siemens Medical Solutions, Erlangen, Germany). A high-resolution T1-weighted whole-brain anatomical scan (1 mm3 voxel resolution, MPRAGE) was acquired before functional imaging. During the task, functional brain images were obtained in 36 axial slices using an echo planar imaging (EPI) pulse sequence, with a TR of 2000 ms, a TE of 30 ms, a flip angle of 90°, a field of view of 240 × 240 mm2, a matrix size of 64 × 64, and a slice thickness of 4 mm with no gaps. The stimuli were presented on a computer screen using fMRI-compatible video goggles (Nordic Neurolab, Bergen, Norway). The participants registered their responses by pressing response grip buttons with their index fingers (Nordic Neurolab, Bergen, Norway).

### Imaging data analysis

Image preprocessing and statistical analyses were performed using SPM8 (http://www.fil.ion.ucl.ac.uk/spm). Functional images were realigned to the first volume to correct for head motion, spatially normalized to a standard stereotaxic space (Montreal Neurological Institute, MNI), resampled to 3 mm isotropic voxels, and smoothed using a Gaussian kernel with a full-width at half-maximum of 6 mm. Realignment parameters were inspected to identify any participants with excessive head movement. Statistical data analyses were performed using a general linear model approach. The durations of the second and third sentences for each scenario were modeled as events convolved with the canonical hemodynamic response function. The first-level design matrix included four regressors of interest corresponding to the empathy scenario from the patient’s perspective (empathy_patient), neutral scenario from the patient’s perspective (neutral_patient), empathy scenario from the doctor’s perspective (empathy_doctor), and neutral scenario from the doctor’s perspective (neutral_doctor). The durations of the first sentence, rating phase, and six movement parameters were entered as regressors of no interest.

After model estimation, three linear contrasts were defined: all empathy conditions > all neutral conditions, empathy_patient > neutral_patient, and empathy_doctor > neutral_doctor, each resulting in a *t* statistic for each voxel. These contrast images were subjected to second-level analyses. Initially, brain regions involved in empathy were determined using group analysis, according to a random effect model. A one-sample t test was performed with the all empathy > all neutral contrast, yielding a statistical parametric map of brain regions sensitive to empathy processing. Multiple statistical comparisons were corrected using Monte Carlo simulations conducted in the AFNI program 3dClustSim (version 18.0.05), with an initial cluster-forming single-voxel threshold of *p* < 0.005 (uncorrected), within a grey matter brain mask thresholded at 40% intensity, yielding a minimum cluster size threshold of 92 voxels (2484 mm^3^) to achieve a whole-brain-corrected cluster-wise threshold of *p* < 0.05.

Secondly, a whole brain voxel-wise mixed ANOVA analysis was conducted with perspective (patient vs. doctor) as a within-subject factor and group (nonmedical vs. medical student) as a between-subject factor. Linear contrasts of [empathy_patient > neutral_patient] and [empathy_doctor > neutral_doctor] were entered as the units of analysis. The main effect of perspective and group indicated brain regions showing differences in empathy-related functional activation depending on perspectives and group category. The perspective by group interaction indicated brain regions showing differences in the effect of perspective across the nonmedical and medical student groups. The ANOVA results were masked inclusively by the all empathy > all neutral contrast, in order to focus on differences in brain activity in regions associated with empathy processing. Statistically significant activations were set at a combined threshold of *p* < 0.005, uncorrected, and a minimum cluster size of 23 voxels (621 mm^3^), corresponding to a corrected familywise error rate (FWE) of *p* < 0.05, as determined by the AFNI program 3dClustSim (version 18.0.05). Finally, eigenvariates were extracted from the activation clusters and plotted to examine the direction of the observed effect (Friston et al., 2006).

Next, we conducted second-level moderated regression analysis to examine group differences in the association between trait-level empathy and the neural activity underlying empathic responses toward the patient and the doctor. The interaction terms were calculated by multiplying IRI trait empathy scores and a group factor (medical students coded as 1 and nonmedical students coded as –1). Group × affective empathy and group × cognitive empathy interactions were entered as regressors in the model, with group, affective empathy, and cognitive empathy as regressors of no interest along with each participant's contrast image of [empathy_patient – neutral_patient] and [empathy_doctor – neutral_doctor], respectively. Activation results were masked inclusively by the all empathy > all neutral contrast and statistical significance was set at a combined threshold of *p* < 0.005, uncorrected, and a minimum cluster size of 23 voxels (621 mm^3^), as determined by the AFNI program 3dClustSim to achieve a corrected corrected familywise error rate (FWE) of *p* < 0.05. Brain activations that covaried with the interaction term reflected group differences in the association between trait empathy and neural responses of empathy. The first eigenvariate was extracted from each activation cluster and was plotted to examine how the association differed depending on the group factor.

## Results

### Behavioral data

Descriptive statistics for each group and group comparison results are presented in Table 1. No group differences were found in either age or state and trait measures of anxiety and depression. Participants’ positive and negative affect scores, as measured by PANAS, were also comparable. Individual differences in empathy, as measured by IRI, revealed between-group differences. Medical students showed significantly less affective empathy (consisting of empathic concern and personal distress subscales), *t* (36) = 2.55, *p* = 0.015 and cognitive empathy (consisting of perspective-taking and fantasy subscales), *t* (36) = 2.49, *p* = 0.018 compared with nonmedical students.

**Table 1:** The means and standard deviations of age, STAI, BDI-II, PANAS, and IRI scores for each group and statistical differences between groups

Group	Medical Students (n = 19)		Nonmedical Controls (n = 19)		Statistics	
	Mean	SD	Mean	SD	*t*	*p*
Age	24.53	1.71	23.74	2.08	1.28	0.209
STAI						
STAI-S	45.63	1.80	45.00	1.76	1.09	0.282
STAI-T	47.26	2.13	48.26	3.03	1.18	0.247
BDI-II	2.63	2.81	4.00	2.91	1.48	0.149
PANAS						
Positive affect	17.89	3.45	18.11	4.57	0.16	0.874
Negative affect	20.47	5.19	18.42	4.74	1.27	0.211
IRI						
Affective empathy	35.79	6.32	41.42	7.26	2.55	0.015
Cognitive empathy	40.26	4.90	44.84	6.34	2.49	0.018

*STAI* Spielberger State-Trait Anxiety Inventory, *STAI-S* state version of STAI, *STAI-T* trait version of STAI, *BDI-II* Beck Depression Inventory II, *PANAS* positive affect and negative affect scale, *IRI* the interpersonal reactivity index

Self-reported ratings obtained during the empathy task are presented in Table 2. Statistical analyses were conducted on ratings of emotional valence, empathic concerns, and perspective-taking success. Ratings specific to empathy scenarios were assessed by subtracting ratings for neutral scenarios from ratings for empathy scenarios. These differential rating data did not meet normal distribution requirements for parametric testing; therefore we used the Aligned Rank Transform (ART; Wobbrock et al., 2011) implemented in the ARTool (version1.6.2, Washington USA; https://depts.washington.edu/acelab/proj/art/) for statistical analyses. An ART relies on a preprocessing step that ‘aligns’ data before applying averaged ranks, making the non-parametric data suitable for ANOVA (see Wobbrock et al., 2011 for a more detailed description). Thus, results from ART are interpreted similarly to the ANOVA results. We conducted two-way ANOVAs on aligned rank transformed differential rating data with perspective (patient versus doctor) as a within-subject factor and group as a between-subject factor. We employed Mann–Whitney U tests for post-hoc comparison tests.

**Table 2:** Self-reported ratings of emotional valence, empathic concern, and perspective-taking success for each group

Group	Medical Students (n = 19)						Nonmedical Controls (n = 19*)						Statistics
Scenario	Empathy		Neutral		Diff		Empathy		Neutral		Diff		
	M	SD	M	SD	M	SD	M	SD	M	SD	M	SD	
*Valence (− 7, very positive; + 7, very negative)*													
Patient	4.29	1.08	− 2.11	1.07	6.40	1.68	3.85	1.15	− 1.99	1.39	5.83	2.15	**P**: *F* = 114.34, *p* < .001
													**G**: *F* = 1.52. *p* = .226
Doctor	2.69	1.63	− 1.34	1.09	4.04	2.01	2.06	0.85	− 1.30	1.05	3.36	1.77	**P × G**: *F* = 0.03, *p* = .861
*Empathic Concern (1, not at all; 7, very much) *													
Patient	5.54	0.64	3.04	1.51	2.50	1.24	5.50	0.75	3.05	1.64	2.46	1.29	**P**: *F* = 147.73, *p* < .001 **G**: *F* = 0.19, *p* = .669 **P × G**: *F* = 0.99, *p* = .326
Doctor	2.66	1.13	2.47	1.68	0.19	1.78	2.25	1.22	2.25	1.48	0	1.25	
*Perspective-taking Success (1, not at all; 7, very well) *													
Patient	5.75	0.64	5.35	0.82	0.39	0.65	5.68	0.86	5.39	1.21	0.29	0.74	**P**: *F* = 11.44, *p* = .002 **G**: *F* = 4.43, *p* = .043 **P × G**: *F* = 3.08, *p* = .088
Doctor	5.54	0.64	5.33	0.88	0.21	0.57	4.63	1.24	5.13	1.42	− 0.50	1.31	

Diff. indicates differential rating scores calculated by subtracting ratings for neutral scenarios from ratings for empathy scenarios. Statistics resulted from two-way ANOVAs on the differential ratings after aligned rank transform (ART)

P = Perspective; G: Group; P × G = Perspective × Group

*One nonmedical student participant was excluded from the statistical analyses on empathic concern and perspective-taking success due to the technical failure of recording post-scan rating data

**Fig. 2:**
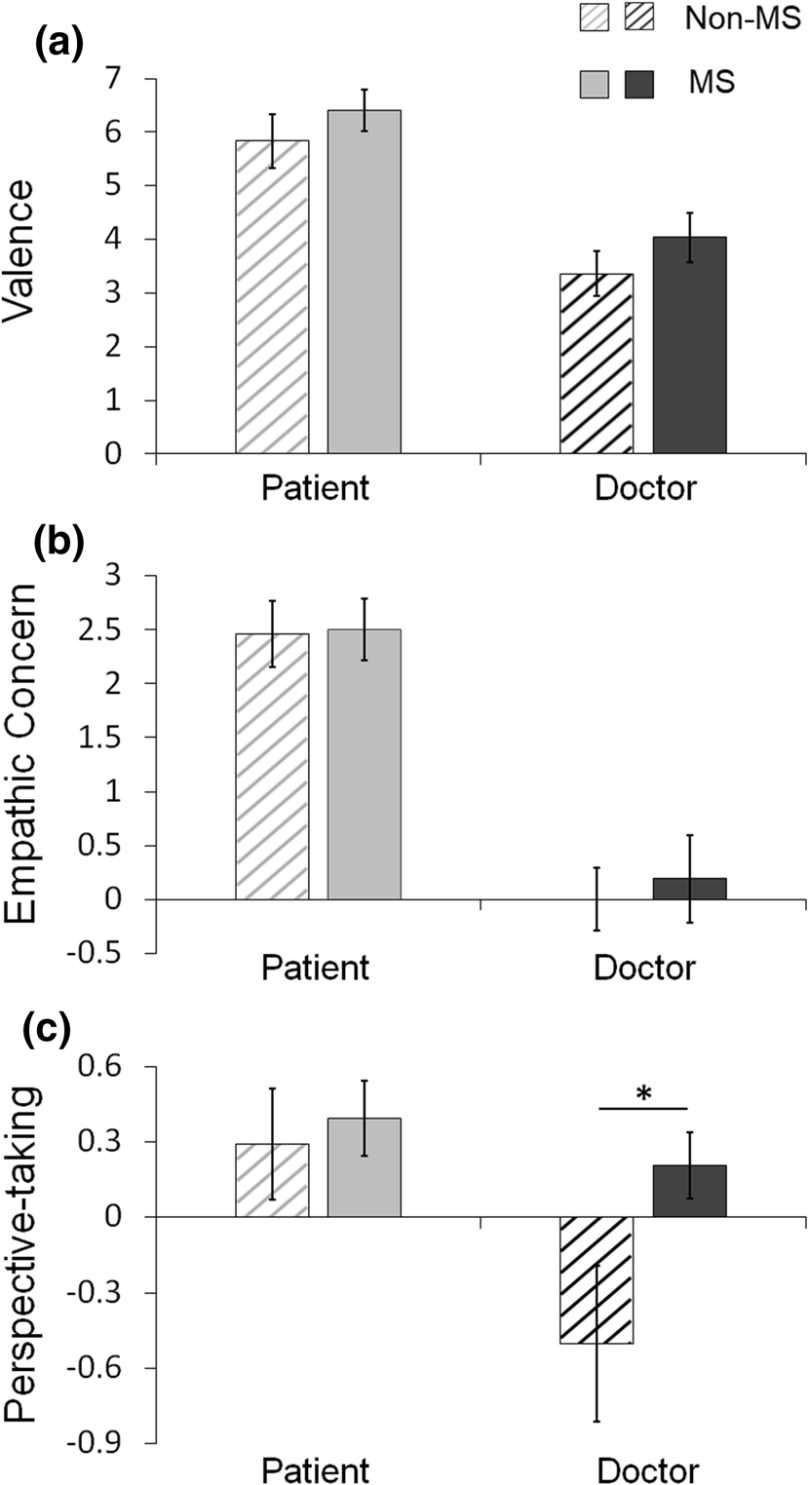
Differential self-reported ratings of empathy vs. neutral scenarios. **a** Emotional valence, **b** Empathic concern, and **c** Perspective-taking success. Error bars indicate the standard errors of the means. MS, medical students; non-MS, nonmedical students. * *p* < .01 (Mann–Whitney)

For the differential ratings of emotional valence, the ANOVA revealed a main effect of perspective, *F* (1, 36) = 114.34, *p* < 0.001. Overall, both groups reported increased negativity under the patient perspective condition (*M* = 6.12, *SD* = 1.37) than the doctor perspective condition (*M* = 3.70, *SD* = 1.34). There was no main effect of group or interaction effect, *F*s < 1.53, *p*s > 0.22 (Fig. 2a). For the differential ratings of empathic concern, similar to the valence ratings, the ANOVA revealed a main effect of perspective, *F* (1, 35) = 147.73, *p* < 0.001. Overall, both groups reported increased empathic concern under the patient perspective condition (*M* = 2.48, *SD* = 1.36) than the doctor perspective condition (*M* = 0.10, *SD* = 1.65). There was no main effect of group or interaction effect, *F*s < 1.00, *p*s > 0.32 (Fig. 2b). For the differential ratings of perspective-taking success, the ANOVA revealed a main effect of perspective, *F* (1, 35) = 11.44, *p* = 0.002, and group, *F* (1, 35) = 4.43, *p* = 0.043. Overall, both groups reported greater success in perspective-taking under the patient perspective condition (*M* = 0.34, *SD* = 0.70) than the doctor perspective condition (*M* = − 0.15, *SD* = 1.02), and medical students (*M* = 0.30, *SD* = 0.76) reported greater perspective-taking success than nonmedical students (*M* = − 0.11, *SD* = 0.78). The interaction effect was marginally significant, *F* (1, 35) = 3.08, *p* = 0.088. Follow-up tests indicated that the medical students reported greater success at taking the doctor perspective (*M* = 0.21, *SD* = 0.57) than the nonmedical students (*M* = − 0.50, *SD* = 1.31), *U* = 86.5, *p* = 0.009; no differences between the groups were observed for the patient perspective condition (medical *M* = 0.39, *SD* = 0.65; nonmedical *M* = 0.29, *SD* = 0.74), *U* = 149.5, *p* = 0.518 (Fig. 2c).

### Functional MRI data

Brain regions activated in association with empathy scenarios relative to neutral scenarios are summarized in Table 3. Empathy scenarios relative to neutral scenarios activated brain regions involved in affective empathy, such as the left amygdala, bilateral inferior frontal gyrus (IFG; Brodmann area [BA] 45, BA 47), and left pre- and postcentral gyri (BA 3, BA 4), and in cognitive empathy, such as the medial prefrontal cortex (MPFC; BA 10, BA 8), bilateral middle temporal gyrus (BA 21, BA 22) extending to the TPJ (BA 39) and temporal pole (BA 20), bilateral precuneus (BA 23), and left hippocampus.

**Table 3:** Brain regions activated when reading empathy-eliciting scenarios versus neutral scenarios

	HEM	BA	Coordinates (MNI)			k (volume)	Z
			x	y	z		
Empathy > Neutral							
Mid. Temporal G. (extending to IFG and TPJ)	R	21	57	− 16	− 11	1371	7.19
Mid. Temporal G	R	21	54	− 31	− 2	(LM)	6.32
Mid. Temporal G	R	21	54	2	− 20	(LM)	6.24
Temporal Pole (extending to IFG and TPJ)	L	20	− 45	11	− 26	1727	6.97
Mid. Temporal G	L	21	− 57	− 22	− 5	(LM)	6.29
Mid. Temporal G	L	22	− 60	− 52	19	(LM)	5.58
Rectus G	L	11	− 3	50	− 17	214	5.77
Med. Orb. Frontal G	R	11	6	53	− 14	(LM)	5.44
Sup. Med. Frontal G	R	10	6	62	28	364	5.51
Sup. Med. Frontal G	R	32	6	53	22	(LM)	5.08
Sup. Med. Frontal G	L	10	− 3	53	28	(LM)	4.29
Precuneus	R	23	6	− 58	28	290	5.49
Precuneus	L	7	− 9	− 55	40	(LM)	4.08
Precuneus	R	7	0	− 52	40	(LM)	3.92
Postcentral G	L	3	− 51	− 22	55	352	5.47
Precentral G	L	4	− 39	− 19	55	(LM)	5.33
Precentral G	L	6	− 33	− 16	70	(LM)	5.28
Amygdala	L		− 21	− 7	− 14	195	4.89
Hippocampus	L		− 20	− 10	− 14	(LM)	4.46
Thalamus	R		6	− 4	− 11	(LM)	3.52
Sup. Med. Frontal G	R	8	9	32	61	152	4.30
Sup. Med. Frontal G	L	8	− 6	32	61	(LM)	3.84
Supp. Motor Area	R	6	6	14	67	(LM)	3.81

*LM* local maxima for activation clusters, *HEM* hemisphere, *L* left, *R* right, *BA* Brodmann area, *k* volume in voxel units, *Z* maximal Z score for contrast, *Mid*. Middle, *Sup.* Superior, *Med.* Medial, *IFG* inferior frontal gyrus, *Orb.* Orbital, *Supp.* Supplementary, *G.* gyrus

Clusters survived a corrected family wise error rate of *p* < 0.05, defined by Monte Carlo simulations conducted in the AFNI program 3dClustSim (*p* < 0.005 uncorrected, k = 92)

**Fig. 3:**
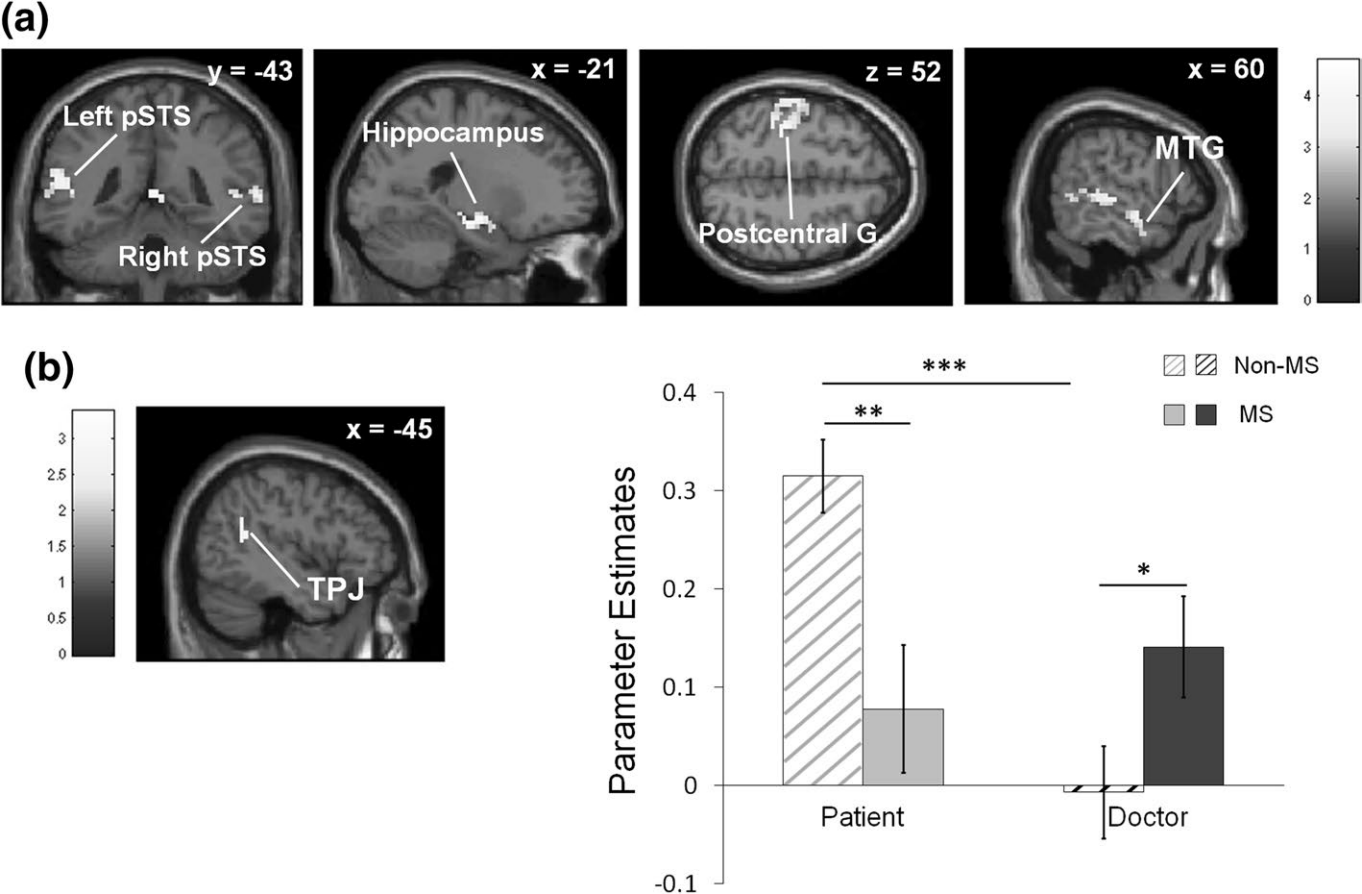
Brain responses associated with empathy. **a** Main effect of perspective (patient > doctor) across the group conditions. **b** A significant group $\times$ perspective interaction emerged in the left TPJ. pSTS, posterior superior temporal sulcus; G., gyrus; MTG, middle temporal gyrus; TPJ, temporo-parietal junction; MS, medical students; non-MS, nonmedical students. * *p* < .05, ** *p* < .01, *** *p* < .001

As shown in Table 4, the whole brain voxel-wise ANOVA revealed the main effect of perspective in such a way that taking the patient perspective, relative to the doctor perspective, elicited greater activation in regions including the bilateral pSTS (left: BA 37; right: BA 21), left postcentral gyrus (BA 4), left amygdala, and right middle temporal gyrus (BA 21; Fig. 3a). No regions were activated for the reverse contrast. An interaction effect of perspective and group was identified in the left TPJ (BA 39; Fig. 3b). Follow-up *t* tests on eigenvariate values extracted from this interaction cluster revealed that activity in the left TPJ was reduced during the patient perspective condition and increased during the doctor perspective condition, among medical students relative to nonmedical students.

**Table 4:** A summary of brain regions showing the main and interaction effects of perspective and group

	HEM	BA	Coordinates (MNI)			k(volume)	Z
			*x*	*y*	*z*		
Perspective × group interaction							
(Non-MS_Pat > Non-MS_Doc) > (MS_Pat > MS_Doc)							
Angular G. (TPJ)	L	39	− 42	− 52	39	23	3.25
Sup. Temporal G	L	41	− 48	− 43	19	(LM)	3.21
(MS_Pat > MS_Doc) > (Non-MS_Pat > Non-MS_Doc)							
No activation							
Group Main Effect							
Non-MS > MS							
No activation							
MS > Non-MS							
No activation							
Perspective Main Effect							
Patient > Doctor							
Hippocampus	L		− 21	− 19	− 14	28	4.29
Mid. Temporal G. (pSTS)	R	21	54	− 52	4	141	4.25
Sup. Temporal G	R	21	57	− 25	1	(LM)	4.09
Sup. Temporal G	R	48	48	− 22	− 2	(LM)	3.94
Mid. Temporal G. (pSTS)	L	37	− 51	− 64	13	345	4.08
Mid. Temporal G	L	21	− 51	− 55	10	(LM)	4.07
Mid. Temporal G	L	22	− 63	− 10	− 5	(LM)	3.94
Postcentral G	L	4	− 36	− 25	52	239	3.95
Postcentral G	L	2	− 39	− 37	55	(LM)	3.91
Postcentral G	L	4	− 45	− 19	43	(LM)	3.75
Mid. Temporal G	R	21	60	− 7	− 14	25	3.34
Mid. Temporal G	R	21	60	− 1	− 23	(LM)	2.80
Doctor > Patient							
No activation							

*Note*. LM, local maxima for activation clusters; HEM, hemisphere; L, left; R, right; BA, Brodmann area; k, volume in voxel units; Z, maximal Z score for contrast; MS, medical students; Non-MS, nonmedical students; TPJ, temporo-parietal junction; Sup., superior; pSTS, posterior superior temporal sulcus; Mid., middle; G., gyrus. Clusters survived a corrected family-wise error rate of *p* < 0.05, defined by Monte Carlo simulations conducted in the AFNI program 3dClustSim (*p* < 0.005 uncorrected, k = 23)

**Fig. 4:**
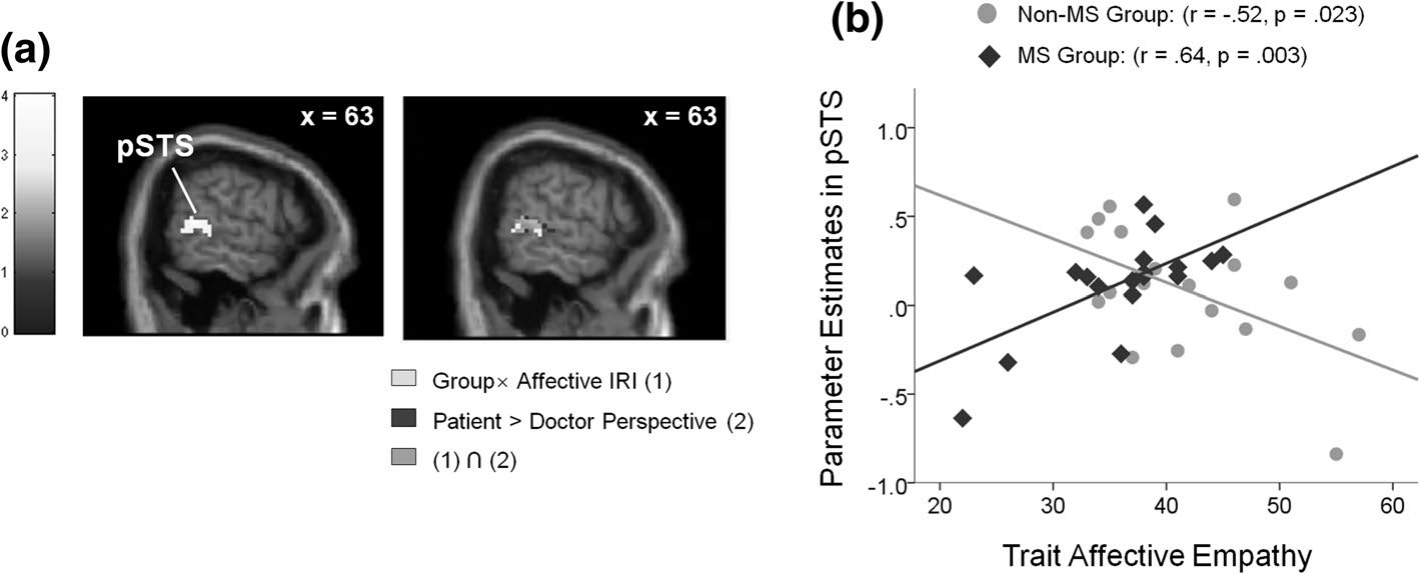
**a** The moderated regression revealing pSTS activity under doctor perspective-taking predicted by the interaction between group and self-reported affective IRI (left). The overlapping pSTS cluster showing the main effect of perspective (patient > doctor) in ANOVA analysis (right). **b** A scatterplot illustrating empath-related activity in the pSTS during the doctor condition as a function of affective IRI in the medical (MS) and nonmedical student (non-MS) groups. pSTS, posterior superior temporal sulcus; IRI, Interpersonal Reactivity Index

Moderated regression analysis revealed that the interaction between group and self-reported IRI affective empathy significantly predicted activity in the right pSTS under the doctor perspective condition (Fig. 4a). Figure 4b illustrates the association between pSTS activity and self-reported affective empathy scores, which revealed a significant positive correlation in medical students (*r* = 0.637; *p* = 0.003) and a significant negative correlation in nonmedical students (*r* = − 0.518; *p* = 0.023). Brain responses were neither predicted by group $\times$ affective empathy interactions under the patient perspective condition nor by group $\times$ cognitive empathy interactions under both perspective conditions (Table 5).

**Table 5:** Brain regions showing the interaction effect of IRI empathy scores and group

	HEM	BA	Coordinates (MNI)			k (volume)	Z
			*x*	*y*	*z*		
*Group* × *Affective Empathy Interaction during Doctor Perspective*							
Mid. Temporal G	R	22	63	− 43	7	46	3.58
Mid. Temporal G	R	21	66	− 49	1	(LM)	2.96
*Group* × *Affective Empathy Interaction during Patient Perspective*							
No activation							
*Group* × *Cognitive Empathy Interaction during Doctor Perspective*							
No activation							
*Group* × *Cognitive Empathy Interaction during Patient Perspective*							
No activation							

*IRI* interpersonal reactivity index, *LM* local maxima for activation clusters, *HEM* hemisphere, *L* left, *R* right, *BA* Brodmann area, *k* volume in voxel units, *Z* maximal Z score for contrast, *Mid.* Middle, *G.* gyrus

Clusters survived a corrected family-wise error rate of *p* < 0.05, defined by Monte Carlo simulations conducted in the AFNI program 3dClustSim (*p* < 0.005 uncorrected, k = 23)

## Discussion

The current study aimed to examine the differences in behavioral and neural measures of empathic responses between medical and nonmedical students and how these responses were altered by adopting different perspectives. Self-reported trait measures of affective and cognitive empathy scores as assessed by the IRI questionnaire were reduced in medical students relative to nonmedical students, which is consistent with previous self-report studies of empathy in medical trainees (Hojat et al., 2009; Neumann et al., 2011; Newton et al., 2008). However, both medical and nonmedical student groups reported comparable levels of negative emotions and empathic concerns, which were increased while reading the empathy scenarios from the patient perspective than from the doctor perspective. Perspective-taking success under the doctor perspective condition differed across the groups in that the medical students reported greater success than nonmedical students, which is most likely attributable to group differences in personal experience in medicine.

The neuroimaging results showed that overall, empathy scenarios relative to neutral scenarios activated brain regions involved in emotion perception and simulation, such as the left amygdala, bilateral IFG and left sensorimotor region, as well as regions involved in perspective-taking and theory of mind, such as the MPFC, bilateral pSTS, left temporal pole, bilateral precuneus, and left hippocampus. Within this set of activated regions, regardless of group, taking the patient perspective relative to the doctor perspective increased activity in the bilateral pSTS, left postcentral gyrus, and left hippocampus. Importantly, an interaction between group and perspective was found for activation in the left TPJ. Specifically, medical students showed decreased activity in the left TPJ during the patient perspective condition and increased activity during the doctor perspective condition, relative to nonmedical students. Although both the left and right TPJ are key regions involved in inferring the mental states of others (Molenberghs et al., 2016; Schurz et al., 2014), a recent meta-analysis study found that the left TPJ is more selectively activated for mentalizing tasks requiring explicit attention to the mental states of others than implicit engagement in mentalizing, and for tasks presented in verbal narratives than in cartoons and photographs (Molenberghs et al., 2016). Therefore, the left-lateralized interactive TPJ activation appears related to the presentation of tasks based on written scenarios that require explicit attendance to verbally described mental states of patients and doctors. Furthermore, it suggests that medical students are less explicitly engaged in inferring the mental state of patients and more engaged in inferring the mental state of doctors, relative to nonmedical students. The reduced TPJ activation for patients among medical students is a novel finding and contrasts with a previous related study in which medical professionals showed increased activation in the TPJ when observing pain on a familiar person in the hospital context relative to the home context (Cheng et al., 2017). Thus, our results extend previous work by showing that in a similar hospital context, medical students engage neural correlates of empathy differently for patients relative to doctors.

The pSTS, which is neuroanatomically adjacent to the TPJ, showed greater overall activation in the patient perspective condition than the doctor perspective condition for both groups. This functional dissociation between the TPJ and pSTS is notable given that they are often labeled interchangeably (Bzdok et al., 2012) and are commonly activated during the theory of mind and empathy tasks (Patel, Sestieri, et al., 2019b). However, recent rigorous research examining the differential roles of the TPJ and pSTS suggests that the pSTS—located anterior and ventral to the TPJ—is more involved in the domain-general process of attentional reorienting to task-relevant stimuli instead of the domain-specific process of mentalizing, which is specifically mediated by the TPJ (Scholz et al., 2009; Tusche et al., 2016; Young et al., 2010). Adopting the perspective of others during mentalizing requires an attention shift from the self to the other and, therefore, may activate the pSTS as well as the TPJ (Buccino et al., 2007; Decety & Lamm, 2007; Mitchell, 2007). Provided that our scenarios described more sensory and contextual details about the patient than the doctor, and only the patient’s (not the doctor’s) description included personally-identifying characteristics (e.g., an elderly), the pSTS would have been involved to a greater extent in the patient perspective condition, tapping more attentional resources than the doctor perspective condition. The finding of overall greater activation of the left hippocampus and postcentral gyrus in association with taking the patient perspective versus taking the doctor perspective may also be attributable to the greater degree of detail and emotional and contextual information provided in the task about the patients than about the doctors, as these regions are implicated in relational binding of perceptual and contextual information (Eichenbaum, 2004) and representations of the somatosensory properties of emotional information (Damasio, 1998).

Interestingly, self-reported trait affective empathy was associated with activity in the pSTS under the doctor perspective condition, which was moderated by the group factor. Namely, medical students showed stronger pSTS responses as their IRI affective scores increased; whereas nonmedical students showed an association in the opposite direction. This interactive activation can be explained by differences in the perceived attentional salience of distress-related cues described in the empathy scenarios. As we discussed earlier, the pSTS represents attentional reorientation to the task-relevant target (Scholz et al., 2009; Tusche et al., 2016; Young et al., 2010). Increased affective empathy may therefore be associated with a greater tendency to orient toward distress cues as they become more salient (Kang et al., 2017; Wu et al., 2017) and, in turn, engage more attentional resources. Because our empathy scenarios described the patients’ distress in greater detail than that of the doctors, nonmedical students with greater affective empathy may be more inclined toward the patients’ distress and engage fewer attentional resources for the doctors’ distress cues when taking the doctor perspective. For medical students, on the other hand, the perceived in-group familiarity to doctors may have increased the salience of doctor-related distress cues and increased the engagement of attentional resources for those with greater affective empathy. This interactive pattern of pSTS activations was not observed during the patient perspective condition, possibly because patient-related distress cues were very clear and similarly salient across the two groups.

An interesting pattern of differences between groups was identified in the core neural system involved in theory of mind and mentalizing. Mentalizing requires deliberate attempts to reason about others’ mental states based on available perceptual and cognitive information and contributes to cognitive empathy (Shamay-Tsoory, 2011; Singer & Lamm, 2009). Relative to affective empathy, mentalizing is more sensitive to top-down factors, such as executive control (Aboulafia‐Brakha et al., 2011; Bull et al., 2008), perceived social proximity (Krienen et al., 2010), and the motivation to understand the target’s mental state (Ickes, 2011; Klein & Hodges, 2001). Medical students, especially those who transition from preclinical to clinical training and adapt to a professional role while gaining more experience with patients, can strategically adopt “physician empathy” as coping strategies (Hojat et al., 2009). This change may reduce the motivation to ‘get into’ the patients’ mental states, resulting in the recruitment of fewer mentalizing resources. This motivational account of mentalizing reduction among medical students is supported by neural and self-report evidence of successful engagement of mentalizing resources when adopting the doctor perspective. Perceived social proximity to the doctors in the scenario may have increased medical students’ motivation to mentalize with them (Krienen et al., 2010). Our finding of medical students with greater affective empathy showing greater utilization of attentional resources from the pSTS under the doctor perspective condition is consistent with this view.

This study, as one of the first to investigate neural responses related to empathy toward patients, advances our understanding of empathy among medical students. Our findings may have important implications for empathy education during medical training. As empathy is recognized as critical for supporting the doctor–patient relationship and successful clinical outcomes (Decety & Fotopoulou, 2015; Underman & Hirshfield, 2016), efforts have been made to develop effective interventions to cultivate empathy (Batt-Rawden et al., 2013; Bearman et al., 2015). Based on current results, it appears that empathy training in medicine would benefit from an emphasis on the mentalization component of empathy. The mentalizing aspect of empathy (i.e., cognitive empathy) can be taught and is amenable to change by various top-down and psychological factors (Aboulafia‐Brakha et al., 2011; Bull et al., 2008; Ickes, 2011; Klein & Hodges, 2001; Krienen et al., 2010). Therefore, an effective education program to cultivate empathy among medical trainees could consider strategies that maintain and enhance the motivation of medical trainees to understand the patients’ cognitive and affective mental states. Especially during clinical clerkships, teaching interventions that strengthen the ability to gain insight into and increase the awareness of the patient’s concerns and feelings of distress would be helpful. An emphasis on a patient-centered interview for building a therapeutic doctor–patient relationship can reinforce the importance of understanding patient concerns and feelings (Benbassat & Baumal, 2004).

The current findings, however, are limited in that they are correlational in nature and therefore cannot establish a causal relationship between medical training and changes in mentalizing for patients. Also, our empathy task did not elicit activations in the dorsal anterior cingulate cortex and anterior insula. These two regions are essential components of “the pain matrix” and consistently engaged in both the experience and observation of physical pain (Lamm et al., 2011). Although some evidence suggests that psychological distress also elicits activation in the pain matrix (Eisenberger, 2012), a more recent meta-analysis study did not support this view (Vijayakumar et al., 2017). We speculate that the lack of activation in the typical pain matrix regions in the current study might be related to the characteristics of our empathy scenarios. Our empathy scenarios featured a situation in which a doctor’s indifference or lack of empathic care might have increased psychological distress to the patient.

Another limitation regards a potential difference between medical and nonmedical groups. Although both medical and nonmedical groups reported comparable positive and negative affective states at the time of participation, nonmedical students had numerically higher (albeit not statistically significant) depression scores than medical students. Furthermore, we eliminated four participants from the nonmedical student group from data analyses due to head motion inside the scanner exceeding our exclusion criteria. It is unclear whether these differences may have affected the results of the current study. A prospective multi-center study with a large sample size and more information about the participants such as academic performance, burnout levels, and stress resilience, would help derive a clearer understanding of medical students’ empathy.
